# Transcriptome-based analysis of key pathways relating to yield formation stage of foxtail millet under different drought stress conditions

**DOI:** 10.3389/fpls.2022.1110910

**Published:** 2023-02-03

**Authors:** Jing Wang, Zexin Sun, Xinning Wang, Ying Tang, Xinyi Li, Chuanyou Ren, Jingyao Ren, Xiaoguang Wang, Chunji Jiang, Chao Zhong, Shuli Zhao, He Zhang, Xibo Liu, Shuli Kang, Xinhua Zhao, Haiqiu Yu

**Affiliations:** College of Agronomy, Shenyang Agricultural University, Shenyang, China

**Keywords:** foxtail millet, drought stress, physiological index, RNA-seq, DEGs, yield formation

## Abstract

Although foxtail millet, as small Panicoid crop, is of drought resilient, drought stress has a significant effect on panicle of foxtail millet at the yield formation stage. In this study, the changes of panicle morphology, photosynthesis, antioxidant protective enzyme system, reactive oxygen species (ROS) system, and osmotic regulatory substance and RNA-seq of functional leaves under light drought stress (LD), heavy drought stress (HD), light drought control (LDCK) and heavy drought control (HDCK) were studied to get a snap-shot of specific panicle morphological changes, physiological responses and related molecular mechanisms. The results showed that the length and weight of panicle had decreased, but with increased empty abortive rate, and then yield dropped off 14.9% and 36.9%, respectively. The photosynthesis of millet was significantly decreased, like net photosynthesis rate, stomatal conductance and transpiration rate, especially under HD treatment with reluctant recovery from rehydration. Under LD and HD treatment, the peroxidase (POD) was increased by 34% and 14% and the same as H_2_O_2_ by 34.7% and 17.2% compared with LDCK and HDCK. The ability to produce and inhibit O^2-^ free radicals under LD treatment was higher than HD. The content of soluble sugar was higher under LD treatment but the proline was higher under HD treatment. Through RNA-seq analysis, there were 2,393 and 3,078 different genes expressed under LD and HD treatment. According to the correlation analysis between weighted gene coexpression network analysis (WGCNA) and physiological traits, the co-expression network of several modules with high correlation was constructed, and some hub genes of millet in response to drought stress were found. The expression changes relating to carbon fixation, sucrose and starch synthesis, lignin synthesis, gibberellin synthesis, and proline synthesis of millet were specifically analyzed. These findings provide a full perspective on how drought affects the yield formation of foxtail millet by constructing one work model thereby providing theoretical foundation for hub genes exploration and drought resistance breeding of foxtail millet.

## 1 Introduction

Foxtail millet (*Setaria Italic* L.) originated in northern part of China about 8,000 years ago, and is one of the important small Panicoid crops due to the small seeds containing high protein, mineral dietary fiber and antioxidants than the big staple crops, also known as nuricereals (Austin, 2006; Lu et al., 2009). Recent studies shows foxtail millet is of lower glucose release rate which is friendly for daily consumption by diabetic patients in modern society (Muthamilarasan et al., 2016). It is a diploid C_4_ crop with a small genome (~423Mb), less repetitive DNA, short reproductive period, which makes it a model crop for small Panicoid grass crops (Zhang et al., 2012; Kumar et al., 2013). Since the arid and semi-arid condition is the main growth area and in turn is the critical environmental challenge, foxtail millet leaves have evolved synchronously with thick cell walls, small leaf area, neatly arranged epidermal cells, and high water utilization thereby making it climate-resilient to abiotic stresses like salt and drought stress (Choudhary and Padaria, 2015; Rana et al., 2021).

Drought as the main result of global warming and extreme climate change not only has affected the food security but also has already affected sustainable development of agriculture in China. In the past decades, there are tremendous losses caused by drought disaster (Huang et al., 2012). Generally, the phenomenon of drought is caused by a complex of atmospheric, hydrological, and biogeophysical processes (Lu et al., 2019). There are four types of drought, meteorological drought, agricultural drought, hydrological drought and socioeconomic drought in which agricultural drought means a plant cannot be refreshed at critical stage and then result in tremendous reduction of yield or death (Ding et al., 2021). The previous study has shown Jilin, Heilongjiang, Liaoning, Shanxi, Shaanxi and Hebei province, etc., are the main area of agricultural drought disaster by using data processing and analysis (Wang et al., 2019). Moreover, drought disaster has become Northeast-forward in China to hinder plant development, which is the main crop production area (Du et al., 2022). Although, foxtail millet has the ability to resist water deficit to some extent, but it is still been affected both at the seedling stage and peak inflorescence stages; and the direct effect is a serious reduction of yield (Rana et al., 2021).

Drought brings damage mainly from inner biochemical system to outer morphological statue and then disrupts millet production. Studies have shown that the primary injury to plants under drought stress is membrane lipid peroxidation, which leads to cell membrane damage and ultimately accelerates cell membrane disintegration (Liu et al., 2015). Accumulation of reactive oxygen species (ROS) takes the main responsibility of drought-induced membrane damage, which includes hydrogen peroxide (H_2_O_2_), superoxide anion (O^2-^), singlet oxygen (^1^O_2_), hydroxyl radical (-OH), alkoxyl radical (RO), and nitric oxide (NO) (Gollan et al., 2015). Under drought stress, the enzymatic systems activities of superoxide dismutase (SOD), peroxidase (POD) and catalase (CAT) are elevated in foxtail millet, and SOD can convert superoxide anion radicals (O^2-^) into H_2_O_2_ in plant cells while POD and CAT can further scavenge H_2_O_2_ to maintain the level of ROS in cells at a relatively low level, thus reducing the damage caused by drought stress (Alscher et al., 2002; Reddy et al., 2004). The non-enzymatic system ascorbic acid can directly scavenge O^2-^, ^1^O_2_ and -OH and also regenerate tocopherols from tocopheryl radicals, thus providing membrane protection (Smirnoff, 1993; Sofo et al., 2015). Tocopherols eliminates singlet oxygen by charge transfer, glutathione, carotenoids and polyphenols can effectively scavenge peroxyl (Roo-), hydroxyl (-OH) and superoxide anion radicals O^2-^ (Smirnoff, 1993; Millar et al., 2003).

Also, the accumulation of ROS has an effect on photosynthesis in foxtail millet (Gollan et al., 2015; Exposito-Rodriguez et al., 2017). Photosynthesis is a vital life activity in plant growth and development, which has promoted dry matter accumulation and yield formation. The content of ROS in leaves of foxtail millet, mainly produced in chloroplasts, has increased under drought stress, and the excessive accumulation of ROS can oxidize photosynthetic pigments and peroxide chloroplast membrane lipids (Exposito-Rodriguez et al., 2017; Hou et al., 2019), which can cause damage to the cell structure and metabolism, especially to photosynthesis (Cui et al., 2016). Therefore, the ability to maintain photosynthesis under drought stress is an important indicator of drought resistance (He et al., 2016). It is generally believed that the main reason for the photosynthesis decrease in plants under water deficit is stomatal closure, which has blocked the entrance of CO_2_ to impair photosynthetic activity of the chloroplasts (Ma et al., 2016). The drought stress on plants photosynthesis can be mitigated through a series of mechanisms (Gollan et al., 2015; Ma et al., 2016; Exposito-Rodriguez et al., 2017). Osmoregulation is an important tool for plants to alleviate drought stress (Exposito-Rodriguez et al., 2017). When subjected to drought stress, plant cells can accumulate large amounts of metabolic substances such as proline, betaine, soluble sugars, and other osmoregulatory substances to maintain cell expansion pressure to keep the proteins and cell structure stable (Ma et al., 2016). Among them, soluble sugars are one of the drought-induced small molecule solutes, including glucose and sucrose, which are involved in the osmoregulatory effects of plant metabolism and plant protein stability (Lata et al., 2013). Meanwhile, sucrose is not only a form of transport and storage of photosynthetic assimilation and energy (Lata et al., 2013), but also can vitrify the liquid around the chloroplast to reduce the water potential of the cell and resist the adverse environment in drought conditions. As the final products of photosynthesis, starch plays an important role in carbon uptake and plant growth balance, and mainly has accumulated during the photoperiod and supports plant growth during the nocturnal cycle. But under drought stress these starches are re-released as soluble sugars, which are used to participate in plant maintenance of cell expansion and maintain cell integrity (Du et al., 2020).

In recent years, transcriptomics has played a crucial role in elaborating the stress biology, where studies on expression profiling of stress-related genes would be imperatively persuaded. And yet, RNA-seq technology has been widely applied to probe the physiological and biochemical response processes in various crops under abiotic stress, such as sorghum (Abdel-Ghany et al., 2020), rice (Ma et al., 2016), maize (Hayano-Kanashiro et al., 2009; Miao et al., 2017), and cereals (Pan et al., 2018; Xu et al., 2019). Foxtail millet natively with abiotic stress tolerance has a small genome, less repetitive DNA, and a recently released draft genome sequence, which makes thepossibility of its complex molecular biology relating to stress tolerance understood by plant researchers (Bennetzen et al., 2012; Zhang et al., 2012). Using transcriptional analysis, Wang et al. (2017) constructed two comparative RNA-Seq libraries and identified 701DEGs with 72 non-annotated, inferring novel function in common millet at seedling stage while Yadav et al. (2016) identified 55 known and 136 novel miRNAs differentially expressed at seedling stage of foxtail millet. Additionally, fourteen-day-old seedlings leave of foxtail millet after PEG6000 drought treatment were used to find DEGs and identified 24-nt siRNAs regulated genes expressed involving drought and 19 IncRNAs responding to PEG treatment (Qi et al., 2013). Zhang et al. (2019) found drought tolerant cultivars preferred highly stable gene expression models in which the jasmonic acid (JA) signal transduction pathway was one reliable way to drive drought-tolerant mechanism at the seedling stage of Proso millet. Also, at the three-leaf seedling stage of foxtail millet, Xu et al. (2019) monitored that a series of genes relating to transcription factors, channel protein genes, proline and soluble sugar synthesis and ascorbate-glutathione cycle had changed to compromise short-term drought stress.

Up till now, extensive studies have focused on the molecular mechanisms responding to drought stress limiting to the seedling stage but merely pay attention to the seed formation stage which is the key point for final yield. Herein, to mimic extreme changes of drought and precipitation at the reproductive growth stage of foxtail millet, we have investigated the changes of morphological, physiological and biochemical indices under different drought conditions and re-watered conditions and the mechanisms of molecular response of foxtail millet to different drought stress. The results would provide that how photosynthesis, antioxidant protective enzyme system, ROS system, osmoregulatory substance worked together to answer the different droughts stress and re-water; how the final yield of millet effected by different drought conditions and re-watered condition; how the inner molecular mechanisms triggered to response different dehydrations thereby constructing one work model for drought responding of millet at yield formation stage and also provide theoretical reference for drought warning.

## 2 Materials and methods

### 2.1 Materials and experimental design

The test material was “Dajinmiao” widely planted in Liaoning province, and the test was carried out in the rain shelter of the Beishan Scientific Research Base of Shenyang Agricultural University. A total of 12 plots were randomized block design with three replications, namely as light drought stress (LD, 45% field water capacity) and heavy drought stress (HD, 25% field water capacity), light drought control (LDCK, 60% field water capacity) and heavy drought control (HDCK, 60% field water capacity). And each plot was separated by polyvinyl chloride (PVC) waterproof plates with a burial depth of 0.6 m. Water withheld began at booting stage of millet, namely about 50^th^ day after sowing. When the field water capacity of the drought-treated plot reached to 45% and 25%, i.e. 90^th^ day and 104^th^ day after sowing, the relevant index were recorded and determined, and the re-water treatment was done with 60% field water capacity until the harvest.

The experiment had adopted the field planting mode, strip sowing planting, ridge length 2 m, ridge width 0.6 m, leaving 300,000 seedlings • hm^-2^. The fertilization level was the conventional fertilization level (98kgN•ha^-1^, 56kgP_2_O_5_•ha^-1^, 140kgK_2_O•ha^-1^) with traditional field management. The soil moisture analyzer (model: TD-TWB) was buried 20 cm underground to monitor the change of soil moisture in real time. In order to maintain the constant water capacity in the field, several times of irrigation in small amounts was adopted in rehydration of LD and HD, LDCK and HDCK, and irrigation was carried out according to the water content of the soil detected at a depth of 20 cm.

### 2.2 Drought-related indices collection

The net photosynthetic rate, stomatal conductance, and transpiration rate of fully expanded flag millet leaves were measured by TARGAS-1(PP Systems, Amesbury, MA) portable photosynthesizer from 9: 00 a.m. to 12: 00 a.m (Kettler et al., 2022). The fully expanded flag leaf under LD, HD, LDCK and HDCK were taken with three replicates and stored at -80°C. The leaf samples (0.2 g per replicate) were ground using a high-throughput tissue grinder (SCIENTZ-48, Zhuhai Heima instrument company, China), and 2 mL of phosphate buffer solution (PBS, pH=7.8) pre-chilled at 4°C was added, then the tissue homogenate was centrifuged at 13,000 r/min (HEMA TGL-16R Refrigerated Centrifuge, China) 4°C for 15 min. The supernatant was the crude enzyme solution, which was used to determine POD activity (guaiacol method), SOD activity (nitrogen blue tetrazolium colorimetric method) (Yu et al., 2022). 0.1 g leaf samples with three replicates were extracted in 95% ethanol to determine photosynthetic pigment content (ethanol immersion method). In addition, 0.5 g leaf samples with three replicates was extracted to determine proline content (ninhydrin colorimetric method), soluble sugar content (phenol method), and Inhibition and produce superoxide anion radicals (Inhibition and produce superoxide anion assay kit, Nanjing Jiancheng Bioengineering Institute, Nanjing, China), and hydrogen peroxide content (Hydrogen Peroxide assay kit, Nanjing Jiancheng Bioengineering institute, China). During the harvest period, the middle row of each treatment was selected for harvesting and its ear length, single ear weight, 1,000 grain weight and yield were measured.


$$\text{Yield}\left(\text{kg}/{\text{hm}}^{2}\right)=W1\times N1\times N2\times \frac{10000}{L1\times W2}$$

Where W1 is the weight of kernels, N1 is the number of kernels per panicle and N2 is the number of panicles per ridge, L1 is the length of the ridge (m), and W2 is the width of the ridge (m).

### 2.3 RNA extraction, library construction and sequencing

Total RNA of leaves was extracted using the Trizol kit (Invitgen, Carlsbad, CA, USA) according to protocol. RNA-seq libraries were constructed according to the manufacturer’s protocol of the NEBNext Ultra RNA Library Prep Kit for Illumina (NEB#7530, New England Biolabs, Ipswich. MA, USA). RNA sequencing with three biological replications was analysis by using an Illumina Novaseq6000 by Genedenovo Biotechnology Co, Ltd. (Guangzhou, China). After low-quality read removal, the remaining reads were aligned to the reference genome *Setaria italica* V2.0 (www.ncbi.nlm.nih.gov/data-hub/genome) by HISAT(HISAT2).

StringTie 1.3.1 (ccb.jhu.edu/software/stringtie) was used to normalize and estimate gene expression levels in fragments per kilobase of transcript per million mapped reads (FPKM). The FPKM values of triplicate samples were averaged for each gene. Differentially expressed genes (DEGs) were identified based on false discovery rate (FDR)-adjusted P-value ≤ 0.05 and a fold change ≥ 2 or ≤ 0.5 using DESeq2 (Love et al., 2014). Functional annotations of expressed genes were made by the Gene Ontology (GO) database (geneontology.org). All molecular pathways were explored by Kyoto Encyclopedia of Genes and Genomes (KEGG) (www.genome.jp/kegg/).

### 2.4 qRT-PCR

To validate the results of RNA-seq data, 10 genes were selected for quantitative real-time polymerase chain reaction (qRT-PCR). The first strand of cDNA was synthesized using the SYBR Premix Ex Taq kit (TaKaRa, Beijing, China) according to the manufacturer’s instructions. Gene-specifc primers were designed using the Primer-BLAST (GenBank, NCBI), respectively. The gene-specific primers for qRT-PCR were shown in **Table S1**. The primer design was completed by Probegene (Jiangsu, China) and synthesized by Shanghai Shenggong (Shanghai, China). To determine transcript abundances, qRT-PCR was performed with a total volume of 20 μL, containing approximately 10 µL of DNA template, 0.1 µL of each primer (50 µM) and 5 µL of PCR-Mix (2×). All reactions were performed under the following conditions: 10 min at 95°C followed by 40 cycles of 15 s at 95°C, 30 s at 60°C, and melting curves were plotted to confirm PCR specificity. Three biological replicates and three technical replicates were included using the comparative 2^-ΔΔCT^ method (Livak and Schmittgen, 2001) with EF-1α as the internal reference gene (Kumar et al., 2013; Shivhare and Lata, 2016).

### 2.5 WGCNA

WGCNA was performed by the WGCNA R package (WGCNA package v1.69, http://www.genetics.ucla.edu/labs/horvath/CoexpressionNetwork/Rpackages/WGCNA). The raw transcription dataset of all samples was filtered to remove all gene building blocks of FPKM (fragments per thousand bases)<1 to analyze the module’s correlation with physiological data. All gene abundances were normalized. Pearson’s correlation coefficients (PCCs) for each gene-gene comparison were calculated, and an adjacency matrix of the connection strengths was constructed. The best power *β* was optimized to adjust the scale-free property of the co-expression network and the sparsity of connections between genes. Highly similar clusters were merged in the network using the merge Close Modules function using a cutHeight value of 8. Module-trait associations were estimated using the correlation between the module eigengene and the phenotype (PPC, Pvalue), which allows easy identification of the expression set highly associated with the phenotype. Compute module membership (MM) based on the Pearson correlation between expression levels and module characteristic genes was used to determine central genes within modules. A relatively high MM indicated that these genes had relatively high connectivity within the module. Physiological data were correlated with expression data of individual genes with gene significance (GS). The visualization of the co-expression network and the recognition of the hub gene in each module were achieved through Cytosscape software v3.91 (Knights et al., 2022).

### 2.6 Statistical analysis

The analysis of physiological data was evaluated with the analysis of variance procedure test of SPSS Statistics 22.0 software (SPSS Inc., Chicago, IL, USA). A *P* value of 0.05 was considered as a statistically significant threshold value. Graphs were plotted by Origin (OriginPro, Version 2021) and TBtools v1.098761 (Chen et al., 2020).

## 3 Results

### 3.1 Changes in yield of foxtail millet under different drought stress

**Figure 1:**
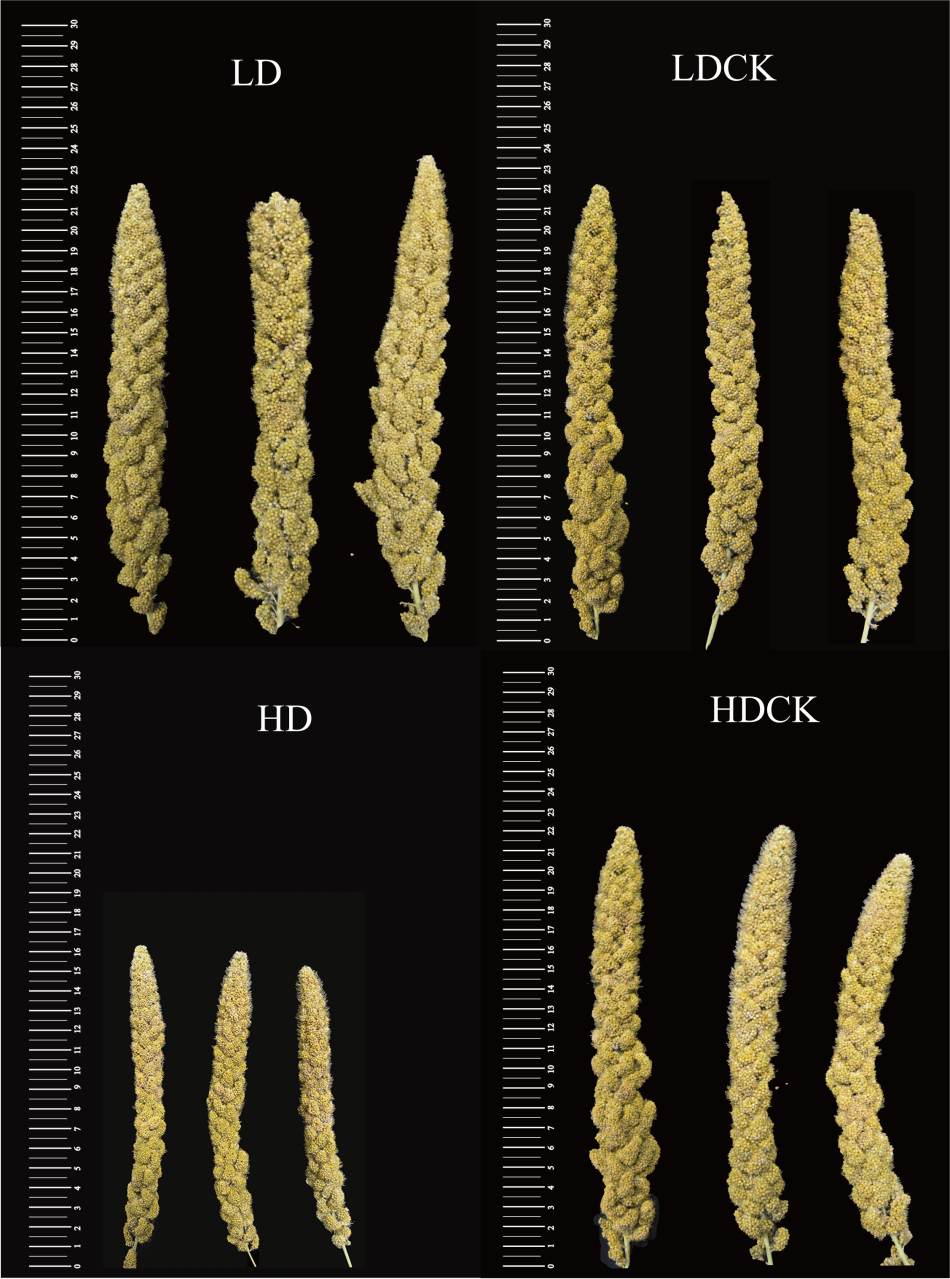
The morphology changes of panicle under different drought treatment and control.

In nature, drought appears randomly and unpredictably and also might be dismissed by timely precipitation. Although foxtail millet is drought-resilient crop, it is the most sensitive to water deficit starting from booting stage to maturity. In order to find damage caused by drought, we mimic different drought stress and rainfall to find out the change of panicle morphology and yield. After LD treatment, the panicles length of millet was 108.9% of LDCK and the number of panicles was 102.6% of LDCK (**Figure 1**), but the panicle weight was 96.8% of LDCK and the weight of 1,000 grains was 94.9% of LDCK. While after HD treatment, the panicles length was shortened by 25.4%, the number of panicles was reduced by 1.6% (**Table 1**), and the panicles weight was reduced by 23.9% compared with HDCK (**Table 1**). Furthermore, the weight of 1,000 grains was 11.4% less than HDCK. In addition, under LD and HD stress, the empty rate of millet increased by 30.8% and 51.5% compared with LDCK and HDCK, respectively. Moreover, drought stress had made significant effects on millet yield which had reduced by 14.9% and 36.9% under LD and HD treatment (**Table 1**), respectively, even though the subsequent rehydration happened.

**Table 1:** Effects of different drought stresses on the panicle and related yield factors of foxtail millet.

	Panicle length (cm)	Panicle number (hm^2^)	Panicle weight (kg/hm^2^)	1,000 grain weight (g)	Empty abortive rate (%)	Production (kg/hm^2^)
LD	23.3 ± 1.34a	301.3 ± 43.2a	3562.4 ± 141.21ab	3.15 ± 0.09a	13.6 ± 0.04b	3725.88 ± 396.76b
LDCK	21.39 ± 1.93a	293.76 ± 28.3a	3678.3 ± 135.6a	3.32 ± 0.15a	10.4 ± 0.02c	4376.4 ± 483.71a
HD	16.43 ± 1.32b	287.13 ± 26.48a	2856.5 ± 318.3b	2.73 ± 0.14b	15.3 ± 0.05a	2785.41 ± 193.63c
HDCK	22.01 ± 1.84a	291.69 ± 21.5a	3751.48 ± 153.39a	3.08 ± 0.13a	10.1 ± 0.03c	4415.28 ± 471.49a

Different lowercase letters show significant difference at the *P*=0.05 level in the same column.

### 3.2 Changes in the physiological characteristics of foxtail millet under drought stress

**Figure 2:**
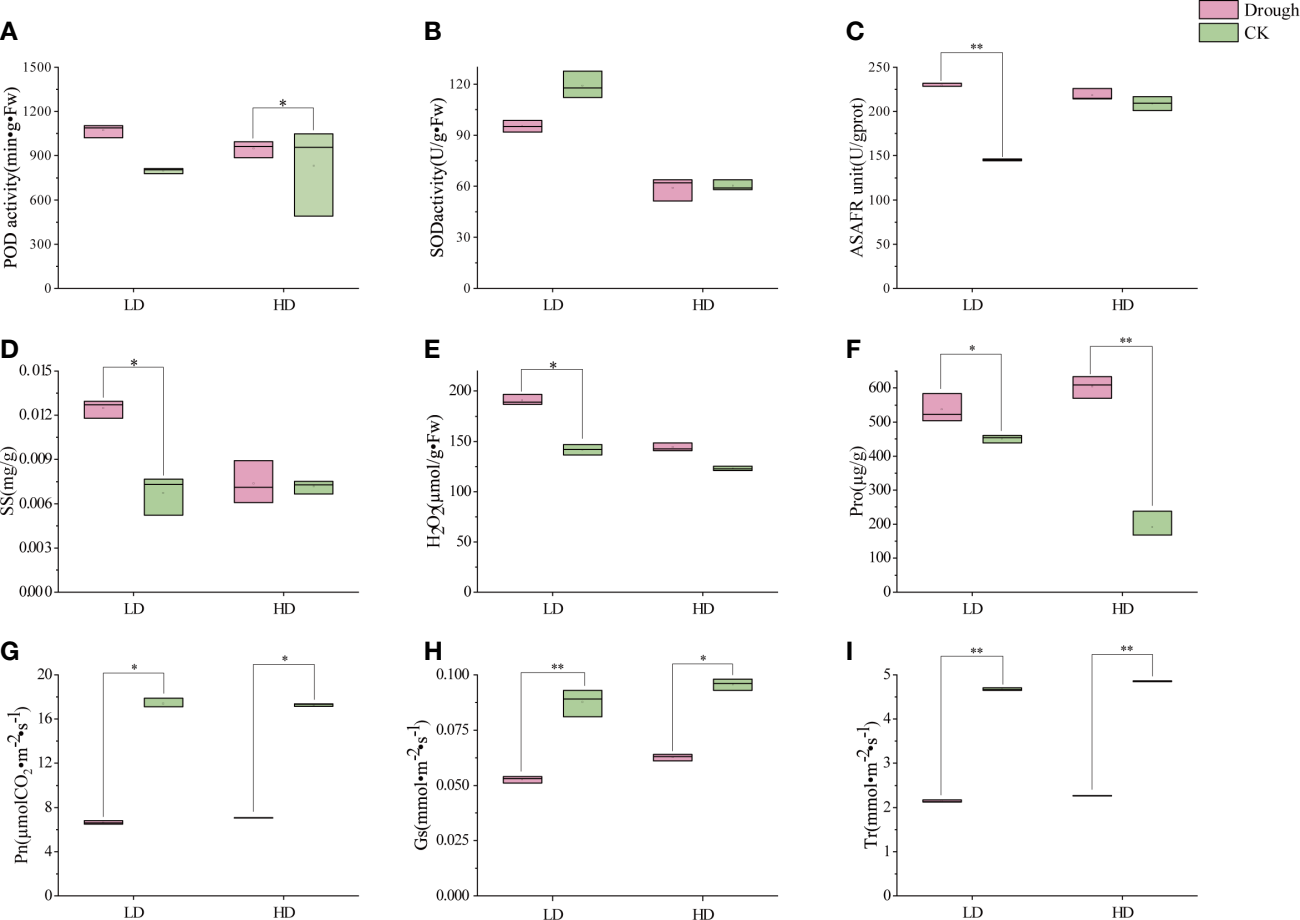
Antioxidant enzyme activities, ROS content, osmotic regulatory substance and photosynthetic characteristics under different drought treatment; **(A)** peroxidase activity (POD); **(B)** superoxide dismutase activity (SOD); **(C)** Anti-superoxide anion viability unit (ASAFR); **(D)** soluble sugars content (SS); **(E)** H_2_O_2_ content; **(F)** proline content (Pro); **(G)** Net photosynthetic rate (Pn); **(H)** Stomatal conductivity (Gs); **(I)** Transpiration rate (Tr). *P<0.05, **P<0.01.

As static creature, plant cannot move or actively avoid from adverse environment. When drought comes, O^2-^free radicals and H_2_O_2_ accumulates, cell membranes and organelles have been harmed, normal cell function is abnormal and then antioxidant enzyme system, permeation regulation has worked to counteract and maintain normal growth and development. POD is an enzyme that cleans up superoxides in plants and can reduce the formation of hydrogen peroxide free radicals within plant cells. Under LD and HD treatment, the POD content in the leaves of millet during reproductive growth increased by 34% and 14% respectively, compared with CK (**Figure 2A**). Meanwhile, SOD content had a slight increase in millet leaves (**Figure 2B**) when millet was subjected to drought stress during reproductive growth (**Figure 2B**). In addition, during the reproductive growth period, LD treatment had a greater impact on the content of hydrogen peroxide in the leaves, and during the LD treatment, the content of hydrogen peroxide in the leaves of the millet was 34.7% higher than that of LDCK, while the HD treatment was 17.2% higher than that of HDCK (**Figure 2E**). The ability to produce and inhibit O^2-^free radicals in LD-treated millet leaves was 58% higher than that of LDCK, while under HD treatment, it was only 4.5% higher than HDCK’s (**Figure 2C**).

Permeation regulation is an important way for plants to adapt to drought adversity, and osmotic regulators, like soluble sugars and proline, actively accumulate when water is scarce. The content of soluble sugars in the leaves of millet during reproductive growth was greatly affected by LD treatment (**Figure 2D**), while the effect was not significantly affected by HD treatment (**Figure 2D**). The content of proline in the leaves of the millet during the reproductive growth period was greatly affected by HD (**Figure 2F**), and the content of proline in the leaves of the HD treated millet was 59.3% higher than that of the HDCK treatment. Pro in LD was 18.9% higher than LDCK (**Figure 2F**).

Under LD and HD, the net photosynthetic rate of millet leaves was significantly reduced (**Figure 2G**). Among them, the inhibition effect of light drought on the net photosynthetic rate of millet is more obvious, and under the light drought stress during reproductive growth, the net photosynthetic rate of millet was only 81% (**Figure 2G**) of 17.4 under LDCK treatment, and 86% of 17.3 under HDCK. Light drought (LD) had a great influence on the stomatal conductance of millet leaves (**Figure 2H**), and under light drought stress, the stomatal conductance of millet leaves was reduced by 40% compared with LDCK, while under HD treatment, the stomatal conductance of millet was reduced by 34% compared with HDCK. For the transpiration rate of millet, the change under LD was consistent with the HD that leads to a 20% reduction in the transpiration rate of millet during reproductive growth (**Figure 2I**). The content of chlorophyll a, b and carotenoids in the leaves of millet under LD treatment had risen; but the content of chlorophyll and carotenoids decreased under HD treatment (**Table 2**).

**Table 2:** Photosynthetic pigments content in foxtail millet leaves under drought stress relating to yield formation stage.

	Ca (mg/g)	Cb (mg/g)	C (a+b) (mg/g)	C (x-c) (mg/g)
LD	0.84 ± 0.1a	0.19 ± 0.03a	1.02 ± 0.13a	0.17 ± 0.01a
LDCK	0.55 ± 0.19ab	0.12 ± 0.04ab	0.67 ± 0.24ab	0.11 ± 0.04a
HD	0.47 ± 0.03b	0.14 ± 0.01b	0.61 ± 0.03b	0.12 ± 0.01a
HDCK	0.71 ± 0.14ab	0.17 ± 0.03a	0.88 ± 0.17a	0.14 ± 0.03a

Ca, Chlorophyll a content; Cb, Chlorophyll b content; C(x-c),Carotenoid content.

Different lowercase letters show significant difference at the *P*=0.05 level in the same column.

### 3.3 Differential expression analysis

**Figure 3:**
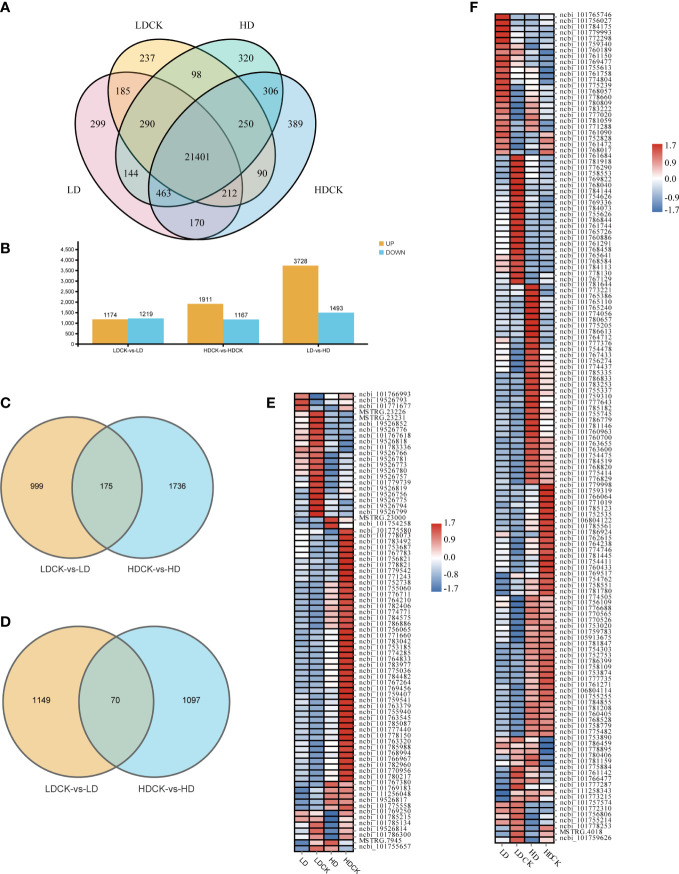
Number of expressed genes with and without simulated drought. The number of genes was selected based on the cut-off values of FDR ≤0.05 and |log_2_ FC|≥ 1. **(A)** Number of genes expressed in each treatment under drought stress. **(B)** Summary of the number of DEGs under drought stress; **(C)** Venn diagrams, representing DEGs including upregulated genes in LDCK and LD and HDCK and HD; **(D)** Venn diagrams, representing DEGs including downregulated genes in LDCK and LD and HDCK and HD; **(E)** Comparison of photosynthesis under drought stress; **(F)** Synthesis of sucrose and starch under genetic drought stress.

To elucidate the transcriptome changes of millet leaves in response to different drought stresses, the RNA-sequencing based transcriptome assay was performed. After data screening and quality control, 0.37% of the low-quality reads were removed, and an approximately clean read of the millet genome was obtained (**Table S2**). Under LD and HD treatment, 22,763 and 23,272 genes were expressed respectively, and 22,763 and 23,281 genes were expressed in LDCK and HDCK, of which 21,401 genes were expressed in different treatments. Under different drought treatments, 22,298 genes were expressed (**Figure 3A**). LD treatment had 2,393 differentially expressed genes during the reproductive growth phase compared to LDCK (**Figure 3A**). Of these, 1,174 genes were upregulated and 1,219 genes were downregulated. There were 3,078 differentially expressed genes under HD treatment, of which 1,911 were upregulated and 1,167 were downregulated (**Figure 3B**). Among them, 175 up-regulated genes and 70 down-regulated genes were synchronously expressed in both LD and HD treatments (**Figures 3C, D**). In addition, with prolonged dehydration, 3,728 genes were detected as up-regulated and 1,493 genes were down-regulated under HD treatment compared to LD treatment.

**Figure 4:**
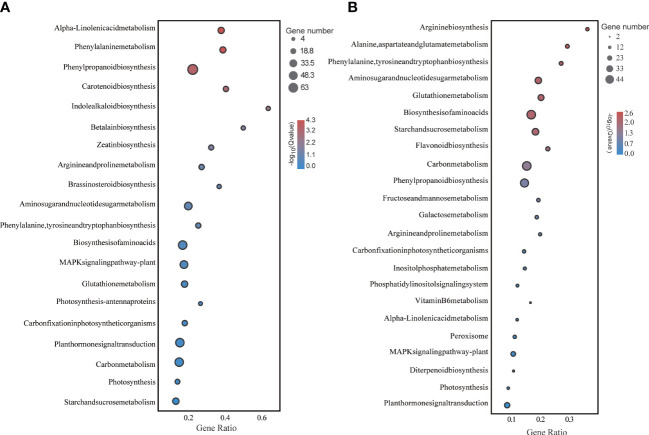
KEGG enrichment analysis of foxtail millet leaves under LD and HD treatment. **(A)** KEGG analysis of differentially expressed genes under LD; **(B)** KEGG analysis of differentially expressed genes under HD.

Functional enrichment analysis of these DEGs under LD and HD showed that they were involved in multiple biological processes. Particularly, photosynthesis and sugar metabolism were more significantly different in both treatments (**Figure 4**).

In addition, a total of 78 photosynthesis-related genes was changed under drought stress in millet. Under HD treatment, the inhibition of photosynthesis-related pathways was more pronounced under HD treatment, with the expression of 63 genes being down-regulated (**Figure 3E**) and only 14 genes being up-regulated. Under LD treatment, the expression of 34 genes was significantly repressed (**Figure 3E**), and the expression of 44 genes was increased. The expression of 141 genes related to sucrose and starch synthesis was changed under drought stress (**Figure 3F**), with the expression of 80 genes rising and 61 genes being repressed under LD treatment. Similarly, there were 76 genes expression levels being significantly increased but 65 genes being repressively expressed under HD treatment when compared to HDCK (**Figure 3F**).

### 3.4 WGCNA analysis of DEGs in millet leaves

**Figure 5:**
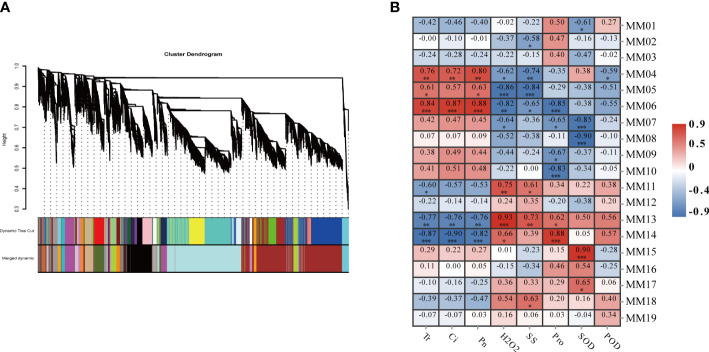
Weighted gene co-expression network analysis (WGCNA) of effectively expressed genes under LD and HD treatment. **(A)** Hierarchical cluster tree showing co-expression modules identified by WGCNA. Each leaf in the tree represents one gene. The major tree branches constitute 19 modules labeled with different colors; **(B)** Correlation analysis between gene co-expression network modules and physiological indices. The horizontal axis represents different physiological traits, and the vertical axis represents the module eigengenes in each module. Each frame contains the corresponding correlations and P values. *P<0.05, **P<0.01, ***P<0.001.

In order to fully understand the closely related gene regulatory network of millet under drought conditions, a scale-free co-expression network based on β=8 soft threshold capacity was constructed by removing the low FPKM (FpKm<1) genes. By WGCNA analysis, while setting the ickHeight to 0.23, clusters of genes with a high degree of inter-association were defined as modules, and genes in the same module were more relevant. Nineteen different modules were always identified by dynamic tree cutting (**Figure 5A**) with module sizes ranging from 50 to 5,329. The MM07 and MM08 modules were the largest, containing 5,329 and 4,782 genes, respectively. The MM19 module had the fewest number of genes.

To identify biologically significant co-expression modules, correlation analysis was performed between the 19 gene co-expression modules and the eight physiological and photosynthetic trait physiological indicators mentioned above. The correlation analysis revealed that MM04 (R2 = 0.76, P<0.001), and MM06 (R2 = 0.86, P<0.01) were positively correlated with photosynthesis, and MM14 (R2 = 0.86, P<0.01), and MM13 (R2 = 0.76, P<0.001) were positively correlated with the indicators of photosynthesis. In terms of physiological traits, the MM13 module was positively correlated with physiological traits and MM06 was negatively correlated with physiological traits. These results suggested that genes clustered in one module are expressed in a similar pattern to resist drought stress. In addition, the MM13 module was positively correlated with MM14 and H_2_O_2_ content (**Figure 5B**) and negatively correlated with photosynthetic properties and. Overall, a total of five co-expression modules (MM06, MM14, MM04, MM13, and MM05) were significantly correlated with specific physiological changes.

**Figure 6:**
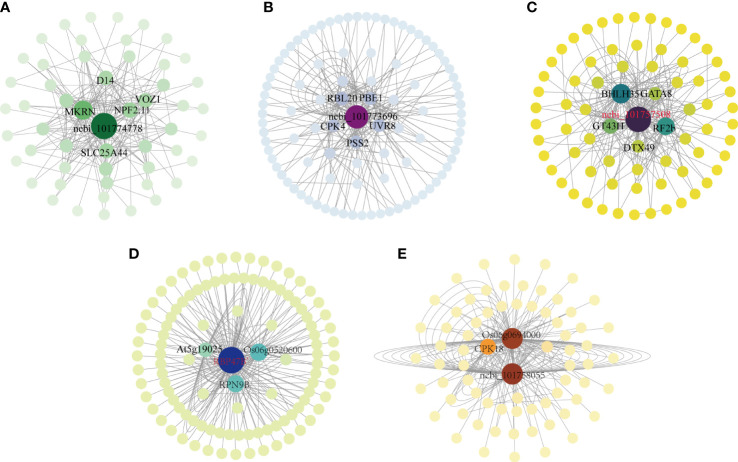
Co-expression regulatory network analysis of five key co-expression modules. **(A)** Co-expression regulatory network analysis of the MM06 module; **(B)** Co-expression regulatory network analysis of the MM13 module; **(C)** Co-expression regulatory network analysis of the MM14 module; **(D)** Co-expression regulatory network analysis of the MM04 module; **(E)** Co-expression regulatory network analysis of the MM06 module.

Plant responses to abiotic stress are controlled by thousands of transcribed genes with different functions and different biological pathways that interact to form complex regulatory networks. Central genes are those that are highly associated with other genes in the regulatory network. Since these genes are located at the center of each module, these genes are thought to play a key role in specific physiological processes (Luo et al., 2019). The association analysis of the differentially expressed genes in the above five modules was carried out, and the 200 gene pairs with the highest correlation were selected to construct a co-expression network map (**Figure 6**), the gene with the highest correlation is used as the hub gene. The hub gene was selected in the MM14 module, the hub gene included a gene encoding the transcription factor bHLH35, which synthesized the possible glucuronyltransferase GT43H with the transcription factor GATA8, and these Hub genes may negatively regulate photosynthesis in foxtail millet (**Figure 6C**).In the MM13 module, there was a gene encoding a polyadenylate-binding protein (*RBP47B’*), the gene encoding an L10-interacting MYB domain-containing protein Os06g0520600 with 26S proteasome non-ATPase regulatory subunit 13 homolog B (*RPN9B*) in the negative regulation of photosynthesis (**Figure 6B**). There was an unknown gene in the MM04 module (*101774778*) a gene encoding solute carrier family 25 member 44 (*SLC25A44*), a gene encoding NRT1/PTR family 2.11 protein (*NPF2.11*), a gene encoding *(E3 ubiquitin protein ligase makorin (E3 ubiquitin protein ligase makorin*) (**Figure 6D**), which had a positive regulatory role in enhancing photosynthesis in millet subjected to drought stress, and a gene encoding adagio-like protein 1 (*Os06g0694000*) in the MM06 module, which had a positive role in regulating photosynthesis (**Figure 6E**). The MM05 module contains genes encoding rhodopsin 20 (*RBL20*), gene encoding GDP-mannose 3,5-epi-isomerase 1 (*GME-1*), gene encoding ultraMM06 B receptor (*UVR8*), transcription factor ERF019 involved in ethylene response, gene encoding CDP-diacylglycerol–serine O-phosphatidyltransferase 2 (*PS22*), the gene controlling calcium-dependent protein kinase (*CPK4*), and the unknown gene (*101773696*) played a positive regulatory role in the synthesis of H_2_O_2_ with soluble sugars (**Figure 6A**).

In this study, the pivotal genes are E3 ubiquitin protein ligase interacting with adagio-like protein 1 to participate in photosynthesis and *ERF019* involved in the ethylene signaling pathway. In addition, there are genes of bHLH35 that play an important role in inducing glutamate synthesis and regulating stomatal regulation of photosynthesis, while glutamate is a precursor of proline synthesis; GATA has an important role in gibberellin signaling, while GT43A plays an important role in plant glycolysis and starch synthesis, but VOZ transcription factor is involved in the formation of plant vascular bundles. Therefore, in this experiment, we analyzed the gene expression changes in several pathways of photosynthetic sugar fixation, sucrose and starch synthesis, gibberellin synthesis and degradation, lignin synthesis and proline synthesis in millet plants.

### 3.5 The changes of related pathways of millet leaves under drought stress

**Figure 7:**
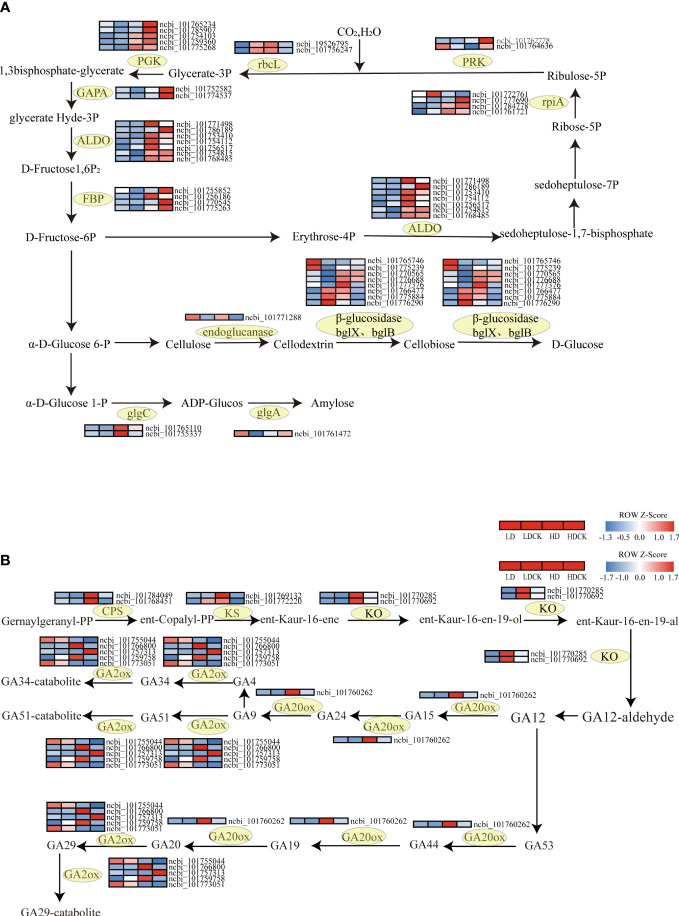
Photosynthetic carbon sequestration with sucrose and starch and synthesis, gibberellin synthesis under drought stress gene changes. The color patch represents the FPKM value. Red and blue indicate significant upward and downward revisions Gene (log_2_ |fold-change| ≥ 1). **(A)** Heat map of drought response DEGs involved in photosynthetic carbon fixation, sucrose and starch synthesis pathways under drought stress. **(B)** Heat map of drought-responsive DEGs involved in gibberellin synthetic pathways under drought stress.

To verify the pathways altered by drought stress, the genetic changes in photosynthesis, sucrose and starch synthesis pathways in millet leaves under drought stress were measured. In the starch and sucrose metabolic pathways, the expression of the gene of the enzyme glgc that controls ADP-glucose synthesis increased to varying degrees under different drought stresses (**Figure 7A**). The expression of the gene that controls the enzyme glgA that synthesizes amylose under HD stress is decreasing, but the expression of glgA-related enzymes is increasing under LD stress. In addition, the expression of the genes *GLU3, BGLU12, BACOVA_02659* and *BGLU14* that control cellulose decomposition under HD treatment was significantly upregulated (**Figure 7A**), the expression of *BGLU30* was significantly downregulated (**Figure 7A**), and the expression of the remaining cellulose decomposition genes were upregulated to varying degrees. The expression of *BACOVA_02659, BGLU12* and *BGLU16* was significantly downregulated under LD stress (**Figure 7A**), and several other genes were significantly upregulated under LD stress (**Figure 7A**). The expression of different genes under different drought treatments showed that millet had different effects on the synthesis of sucrose and starch. The expression of cellulose to glucose was higher under HD stress than LD, and the catabolism of cellulose was higher under HD than LD, and the promotion of glucose synthesis was also greater than LD.

**Figure 8:**
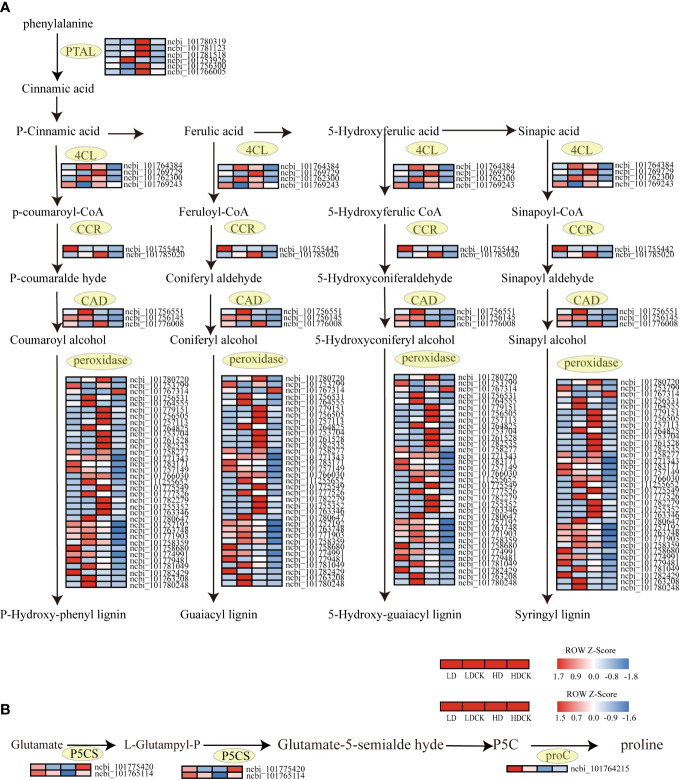
Lignin synthesis, changes in proline synthesis genes under drought stress. The color patch represents the FPKM value. Red and blue indicate Significant upward and downward revisions Gene (log_2_ |fold-change| ≥ 1). **(A)** Heat map of drought response DEGs involved in lignin synthesis pathways under drought stress. **(B)** Heat map of drought response DEGs involved in proline synthesis pathways under drought stress.

In higher plants, proline is synthesized from glutamate mainly by the action of two enzymes: Δ1-pyrroline-5-carboxylic acid synthesis (P5CS) and pyrroline-5-carboxylic acid reductase (P5C). Water deficit-induced proline accumulation may involve the loss of feedback inhibition of P5CS activity by proline and up-regulation of the P5CS gene. In the present study, the expression of P5CS (L-Glutampyl-P), an enzyme that synthesizes L-Glutampyl-P, and P5CS (glutamate-5-semialdehyde dehydrogenase), an enzyme that affects Glutamate-5-semialde hyde synthesis (**Figure 8B**), increased under light drought stress, but expression of these two enzymes in cereal leaves was decreasing in heavy drought stress. These gene changes indicated that LD and HD had differed from the genetic changes in millet that control proline synthesis in the leaves during the panicle stage.

Gibberellic Acid (GA) is mainly stimulated in the growth and developmental overgrowth of plant cells. At early biosynthesis pathway of GA, the three vital enzymes involved, like ent-copalyl diphosphate synthase (CPS), ent-kaurene synthase (KS), ent-kaurene oxidase (KO) were all significantly up-regulated under HD treatment than LD treatment (**Figure 7B**). The expression of genes encoding GA2ox, an enzyme controlling GA degradation, mostly decreased in foxtail millet leaves under LD treatment, but the expression of gene encoding GA20ox, an enzymes involving GA synthesis was up-regulated (**Figure 7B**; **Table S3**). Whilst the genes relating to GA2ox all had significantly up-regulated, the gene encoding GA20ox was slightly up-regulated under HD treatment (**Figure 7B**; **Table S3**).

Lignin is essential for maintaining the integrity of plant vascular tissues to ensure efficient water transport to the tissues that need it most during stress (Malavasi et al., 2016). The genes synthesizing CAD and CCR were upregulated in different drought levels, but the upregulation was greater under HD compared to LD. The expression of genes controlling the synthesis of 4CL and POD increased under HD treatment, while the expression of genes controlling the synthesis of these two substances was decreasing under light drought stress (**Figure 8A**). The changes in these genes indicated that the synthesis of lignin had increased in millet subjected to drought stress, and the accumulation of lignin consistent with drought intensity.

**Figure 9:**
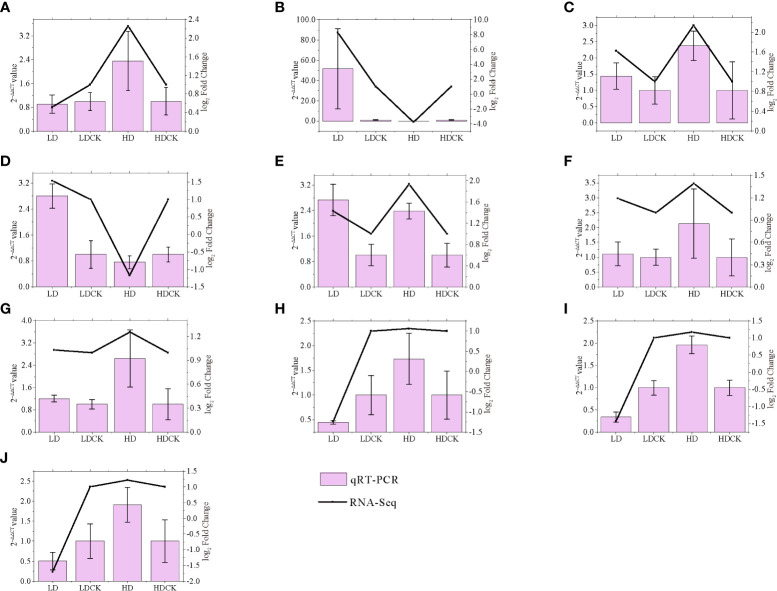
Correlation between qRT-PCR and RNA-seq based on their respective data from twelve candidate transcripts. Each point represents a fold change value of expression at LD or HD compared with that of CK **(A)** AGPL3; **(B)** P5CS2; **(C)** GLU3; **(D)** PSB28; **(E)** ADC2; **(F)** ACS1; **(G)** BGLU7; **(H)** CPA; **(I)** TPP9; **(J)** CIN1.

To validate the gene expression data obtained by RNA-seq, we selected 10 genes involved in photosynthesis, sucrose and starch synthesis, MAPK, and proline synthesis pathways under drought stress for qRT-PCR analysis. We found well agreement in relative gene expression between RNA-Seq and qRT-PCR for all candidate genes under drought stress, which confirmed the reliability and accuracy of RNA-Seq analysis in this study (**Figure 9**).

## 4 Discussion

### 4.1 Regulator mechanism of Lignin biosynthesis under drought stress

When plant grows, the production and removal of reactive oxygen species (ROS) in the body are in a state of equilibrium, a state that has prevented damage to the plant in the event of excessive accumulation of ROS in the body (Reddy et al., 2004). Under drought stress, the membrane system of cells in millet leaves was damaged and an excessive accumulation of ROS was produced. The excessive accumulation of ROS can cause damage to the cellular structure of the plant and a decrease in metabolic function, leading to organ damage, decreased physiological function, and stunted growth and development of the plant (Liu et al., 2015). The results of this experiment showed that the effect of LD treatment on H_2_O_2_ over O^2-^ in millet leaves was greater under drought stress (**Figures 2C, E**). It indicated that the response of millet to drought was more significant under LD treatment. Previous studies had shown that drought stress also inhibited plant photosynthesis by affecting chlorophyll biosynthesis and promoting stomatal closure, leading to the accumulation of malondialdehyde and ROS, which was detrimental to chloroplast photosystem II (PSII) (Demmig-Adams and Adams Iii, 1992; Smirnoff, 1993). Therefore, plants have evolved antioxidant enzyme systems such as superoxide dismutase (SOD), catalase (CAT), and peroxidase (POD) to counteract the damage caused by drought stress (Sharma et al., 2012). Antioxidant protective enzymes had an important role in the scavenging of ROS, which had effectively scavenged the excessive accumulation of reactive oxygen species in cells, reduced the peroxidation process of membrane lipids, and maintained normal plant growth and development (Reddy et al., 2004). In this experiment, the results showed that the content of POD in millet leaves was increased under both LD and HD treatment (**Figure 2A**), and the content of SOD decreased under LD drought stress and with slightly decreased under HD treatment (**Figure 2B**). In order to explore the persistence of H_2_O_2_ content, Anti-superoxide anion viability, POD and SOD, we further investigated what happened after rehydration. The H_2_O_2_ content in millet leaves and the ability to produce and inhibit O^2-^free radicals were restored to LDCK levels at 20 days of rehydration under LD treatment (**Figure S1D**). It inferred that the damage caused by drought had profound effect in plant. The content of SOD had no change after re-water, the same as HD treatment (**Figure S1B**). Whilst under LD treatment, the contents of POD in the millet leaves recovered to LDCK level after 1 day of re-watering, and under HD treatment, it decreased to HDCK level after 3 days of rehydration (**Figure S1A**). Those data showed that under drought stress, POD had played key role in defense, but also LD treatment had worked greater on POD, indicating that the ability of millet to scavenge ROS was stronger under this water content. For the sake of digging molecular mechanism of POD, the function was further investigated. Overexpression of POD have increased the content of phenol and lignin in plants because lignin is usually polymerized with the three main types of monophenols by peroxidase (POD) and lacse (LAC) in the secondary cell wall (Liu Q. et al., 2018). Lignin not only gives land plants rigidity against stress, but also forms a mechanical barrier against pathogens and environmental stresses. The aromatic properties of lignin make cell walls impermeable to water, which was beneficial for plants to reduce transpiration and maintain normal turgor under drought stress (Monties and Fukushima, 2005). As be intensively studied, phenylalanine ammonia-lyase (PAL) and cinnamate-CoA ligase (4CL) have played an important role in lignin biosynthesis by placing at the first and last step of the ‘‘general phenylpropanoid’’ pathway (Lauvergeat et al., 2001). PAL is the primary rate-limiting enzyme to convert L-phenylalanine to trans-cinnamic acid by eliminating ammonia, which has also named PTAL enzyme in monocots (Bai et al., 2021). A multi-gene family expression has involved in PAL enzymes in many plants, and the isoforms might play a distinct but redundant role in responding to plant growth and adverse conditions (Yu et al., 2018). In *Arabidopsis*, only double mutants of *pal1/pal2* have showed significantly decreased lignin cumulating resulting in ultrastructure change of the second cell wall and induced infertility (Rohde et al., 2004). Apart from this, the transcription factor and protein has participated in *PALs* regulation, in which the regulation of transcript level is mainly relating to environment factors, such as R2R3 MYB and KNOX family (Yu et al., 2018). Additionally, the gene expression relating to PAL has been held up by negative feedback of cinnamic acid (Blount et al., 2000). 4CL, as main branch point enzyme, has controlled the biosynthesis of downstream molecule, especially flavonoids and lignins which has often contributed to combat with drought stress (Lavhale et al., 2018). Sun et al. (2020) identified more than 34 genes in *G. hirsutum* and found 26 genes had been induced by multiple stress condition, but one gene of *Gh4CL7* had positive responded to drought stress. In this study, we found that the genes encoding those two important enzymes were both up-regulated under HD treatment but not in LD treatment and hypothetical confirmed that prolonged drought stress had indeed stimulated phenylpropanoid pathway to get more genes in lignin synthesis pathway lighted. Among the genes related to lignin synthesis, there were few reports on the expression of Cinnamoyl CoA reductase (CCR) and lignin deposition under abiotic stresses such as salt and mannitol (Lauvergeat et al., 2001). In many reports, different subtypes of CCR had been reported to perform distinct functions, such as defense, development and stress, of which is played as a control point of monolignol biosynthesis and its activity is positively related with lignin deposition in xylem vessel (Srivastava et al., 2015). In *Arabidopsis*, two genes encoding CCRs (*AtCCRl* and *AtCCR2*) were thought to be differentially expressed (Lauvergeat et al., 2001). And lignification pattern had been occurred in root and stem when seedlings of *Leucaena leucocephala* was committed to drought stress as to the CCR protein was accumulated significantly (Srivastava et al., 2015). Other important lignin biosynthesis gene (*CAD*) was also found to be overexpressed under drought conditions; suggesting the relevance of lignin biosynthesis under abiotic stress conditions (Hu et al., 2009). It played positive correlations with lignin deposition under drought stress (Moura et al., 2010; Li et al., 2013). Liu W. et al. (2018) found five *CmCAD* genes in melon genome database had been positively induced by drought condition. And then the functional analysis showed that those five genes had diversely helped plant get through the drought by recovering lignin synthesis and composition, otherwise the lack formation of tracheary element and Casparian strip had been promoted in silence treatments (Liu et al., 2020). In this experiment, the genes of CAD and CCR were all up-regulated in HD treatment than LD treatment (**Figure 8A**), and it was consistent with the results of Hu et al. (2009) since there was protein spots induced by drought treatment and functioned as CAD, CYP450 and SAMS involving in lignin synthesis. Therefore, it would be valuable to identify the anatomy difference of leave and stem both in LD and HD treatment and to figure out the inner changes of vessel tissue and tracheary element to distract water transport in millet.

### 4.2 Regulator mechanism of carbon fixation and sucrose and starch synthesis under drought stress

Photosynthesis is the basis of plant growth and development, providing material and energy for plant dry matter accumulation and yield formation. Previous studies had shown that drought stress could significantly alter photosynthesis in plants, and persistent drought or severe drought could cause corresponding changes in various physiological and morphological indicators of millet, such as degradation of photosynthetic pigments and chlorophyll, closure of leaf stomata, reduction of intercellular CO_2_ concentration, photosynthetic activity, and disruption of photosynthetic electron system (Lawlor and Cornic, 2002; Zhang et al., 2010), the similar results was also showed up in **Table 1**. Though, as one of the traditional drought tolerant crops in China, millet has survived with yield under drought but it still negatively had an impact on the normal growth and final yield. In the present study, photosynthesis of millet was significantly inhibited under drought stress, and the net photosynthetic rate, stomatal conductance and transpiration rate of millet were more significantly reduced under LD treatment compared with HD treatment (**Figures 2H, I**). Shi et al. (2018) found photosynthetic and transpiration rate of two different genotypes of foxtail millet had significantly decreased under drought treatment, indeed declined in drought-resistant cultivar. Moreover, Cui et al. (2019) found either soil drought condition or PEG treatment had induced reduction of stomatal conductance, transpiration and photosynthetic rate in wheat. The reduction of photosynthesis had led to the reduction of light energy utilization efficiency, and in turn, the excess light energy had excessively accumulated ROS in leaves, which was verified that the H_2_O_2_ content and the ability to produce and inhibit O^2-^ free radicals in millet leaves were restored to LDCK levels until 20 days of rehydration under LD treatment (**Figures S1C, D**). With the accumulation of ROS, HD stress led to the destruction of some functions in the process of photosynthesis and antioxidant protective enzymes, so that each index of which including yield could not be restored by rehydration (**Figures S1C, D**; **Table 1**). The same result was also observed in low light-stress treatment and the similar yield reduction of foxtail millet was occurred (Yuan et al., 2017).

Photosynthesis is a complex process in which photosynthetic electron transfer and the Calvin cycle are key steps, involving the conversion of light energy into ATP and NADPH and the conversion of CO_2_ into carbohydrates. At here, we noticed that the changes in genes in the process of carbon fixation in photosynthesis were more significant in millet under drought stress. Calvin cycle is not only the start point of photosynthetic carbon fixation but also plays vital role in plant metabolism. It consists of three primary stages, carboxylation, reduction, and regeneration and during this process; ATP and NADP are consumed to provide carbohydrate biosynthesis with precursors (Geiger and Servaites, 1994). What’s more, it is GAPA and PGK that consumed ATP and NADP to catalyze glycerate-3-phosphate to form glyceraldehyde-3-phosphate, which is the key enzyme in Calvin cycle (Zhang et al., 2021). *GAPA* and *GAPB* are two genes encoded two subunits of photosynthetic glyceraldehyde-3-phosphate dehydrogenase (GAPDH) and chloroplast localized glyceraldehyde-3-phosphate dehydrogenase which is the key regulator in response to oxidative stress and ABA signal transduction (Sirover, 2011; Li X et al., 2019). Among 11 enzymes involved in Calvin cycle, ALDO as nonregulated enzyme limits photosynthetic rate and restricts carbon flux in CO_2_ fixation because it participates in converting glyceraldehyde 3-phosphate and dihydroxyacetone phosphate (DHAP) to fructose 1,6-bisphosphate and converting erythrose 4-phosphate and DHAP to sedoheptulose 1,7-bisphosphate (Nakahara et al., 2003; Yang et al., 2017). Under LD treatment, the enzymes glyceraldehyde-3-phosphate dehydrogenase (NADP^+^) (phosphorylating) (GAPA), phosphoglycerate kinase (PGK) and fructose-bisphosphate aldolase, class I (ALDO) were elevated (**Figure 9**). However, under HD treatment, not only the expression of genes controlling these enzymes had mostly decreased, but also the expression of fructose-1, 6-bisphosphatase I (FBP), phosphoribulokinase (PRK), and ribulose-bisphosphate carboxylase large chain (rbcL) in the Calvin cycle (**Figure 7**). This indicated that LD treatment had maintained the carbon sequestration process of photosynthesis during the reproductive growth of millet, but HD treatment had partially failed with this process. Since the down-regulated expression level of *GAPA* and *GAPB* would cause accumulation change of soluable sugar and energy production (Ding et al., 2022). The genes expression changes in HD indicated that soluble sugar in chloroplast was decreased and 1,3-diphosphoglycerate supply was inhibited in glycolysis process thereby attenuating photosynthetic carbon fixation. In addition, we also found that the genes encoding to ALDO had up-regulated both under LD and HD treatment. Uematsu et al. (2012) found that overexpression of *AtptAL* in plastid of tobacco had increased plant growth and photosynthesis. But at here, photosynthesis had not been increased and the possible reason was that the expressional regulation or protein degradation had regulated gene expression of ALDO (Graciet et al., 2004). Moreover, the photoelectron transfer efficiency and photochemical efficiency in the leaves of millet had changed, and the structure of the cell had changed to a certain extent, and these results had yet to be explored at next step.

Sucrose in starch is an important substance for plant growth and development, which affects the amount of dry matter accumulated in the plant. Sugar is accumulated in millet leaves during sucrose in starch is an important substance for plant growth and development, and it affects the amount of dry matter accumulated by the plant. Under drought stress, millet leaves accumulate sugar during the elongation phase. As drought stress increases the accumulation of soluble carbohydrates, the carbon requirement for growth arrest is also reduced before the carbon supplied through photosynthesis is reduced (Muller et al., 2011). A similar magnitude of reduction in photosynthetic activity was demonstrated when the early reproductive stages were subjected to drought stress (Muller et al., 2011) (**Figures 2G–I**). In addition to photosynthesis, starch hydrolysis is an important source of soluble sugars, especially in response to stress (Gudesblat et al., 2009). In the present study, the accumulation of soluble sugars in millet leaves was significantly higher under LD treatment than HD treatment. According to the results of RNA-seq, the synthesis of glucose-related synthase in millet leaves was affected under severe drought stress (**Figure 7A**), but glucan synthesis was more affected under LD treatment. In other cereal crops, sugar remobilization from the nutritive part to the spike has been reported to be caused by senescence caused by soil drying (Yang and Zhang, 2006). Under drought stress, plants can inhibit plant growth by reducing photosynthesis, leading to a decrease in sucrose accumulation in plant leaves. Other studies have shown that drought has an effect on the balance of sucrose metabolism in leaves by affecting the activity of sucrose metabolizing enzymes in plant leaves (Pinheiro et al., 2005). Endoglucanase and *β*-glucosidase are important enzymes for the breakdown of cellulose to glucose sugars. Under drought stress, the expression of enzymes used to break down cellulose in plant leaves increases, leading to a decrease in cellulose content and an increase in glucose content. In this experiment, the expression of genes controlling endoglucanase and *β*-glucosidase increased under drought stress, indicating that more cellulose was broken down into glucose under drought stress, which coincided with the increased content of soluble sugars under LD treatment (**Figure 2E**). Consequently, the reduction of starch content in plant leaves under drought stress had promoted the conversion of starch to soluble sugars and then the accumulation of sucrose (Lee et al., 2008; Du et al., 2020).

In the process of re-water treatment, the inhibition effect of drought stress on the net photosynthetic efficiency of millet at the booting stage was restored to LDCK and HDCK level 12 days after rehydration, and the stomatal conductance of millet was greatly affected by drought treatment during reproductive growth and was more difficult to recover (**Figure S1F**). The damage of drought to the transpiration effect of millet cannot be restored if it was rehydrated in a short period of time. These results indicated that even water was re-given after drought; it was inability to restore normal photosynthesis in time. Furthermore, some genes expression or some functional pathway triggered during drought stress was irreversible. Under drought condition, crop growth showed different changes at all levels, including cell, organ, individual and population (Pei, 2014). Wang et al. (2012) found that severe drought stress had the most significant effect on spike quality, grain quality, blight rate and yield of grain during the reproductive growth period, with fearful reduction of yield. Biomass could reflect the growth capacity and nutrient accumulation capacity of a crop throughout its reproductive period (Wang et al., 2007). Drought had caused a highly significant decrease in grain height, spike weight, and thousand grain weights, which was consistent with the results of this study (**Table 1**).

### 4.3 Regulator mechanism of proline biosynthesis under drought stress

The main physiological mechanism of plant adaptation to water stress is osmoregulation. Plants have actively accumulated large amounts of soluble osmotic substances to maintain osmotic balance and protect cell structures during drought (Jiao et al., 2012). Proline (Pro) and soluble proteins are important osmoregulatory substances. Under drought stress, increased osmoregulatory substances have helped to maintain the cytoplasmic expansion pressure and the water potential at a certain level, so that the intracellular physiological and biochemical metabolism has continued. While some osmoregulatory substances represented by Pro have a direct scavenging effect on reactive oxygen species (Zhou et al., 2002; Verbruggen and Hermans, 2008). In this experiment, the free proline content in millet leaves increased differently according to different water capacity (**Figure 2G**). These results confirmed the positive correlation between the degree of drought and Pro accumulation (Dobrá et al., 2011). The increase of Pro in millet leaves under HD treatment was significantly higher than LD treatment (**Figure 2G**). Under abiotic stress, there is a clear-cut positive correlation between Pro accumulation and activity of *Δ^1^-pyrroline-5-carboxylate synthase* (P5CS) since it is rate-limiting enzyme in biosynthesis pathway (Strizhov et al., 1997). Phutela et al. (2000) found P5CS activity under water stress had increased both in leaves and roots of drought tolerant and suspective genotypes, more severe in the former. The similar significantly change of increased P5CS activity in comparison with proliferation of proline level was also appeared both in two different genotypes in cotton (Parida et al., 2008). Goharrizi et al. (2022) identified the gene expression of P5CS and proline content under two levels of drought stress between drought tolerant and susceptive genotypes, and found all the two were both increased significantly, especially in drought tolerant cultivars. Similarly, transforming *P5CS* gene from *vigna aconitifolia* had increased five times more proline content in transgenic chickpea and rice plants than the wild types (Karthikeyan et al., 2011). In general, there are two individual homologous genes of P5CS, *P5CS1* and *P5CS2*, functioned in proline content accumulation, in which *P5CS1* is promoter of flower development and *P5CS2* is key regulator of embryo abortion at the late of seed development (Szekely et al., 2008; Trovato et al., 2008). The *P5CS1* and *P5CS2* activity in leaves under LD treatment was significantly and positively correlated with proline levels, indicating that the accumulation of P5CS was a result of glutamate synthesis, but there was a significant decrease in *P5CS1* and *P5CS2* activity in millet leaves under HD, especially the *P5CS2* (**Figure 8**). In order to further investigate the response mechanism of *P5CS2* gene under drought stress, we later measured proline in millet leaves after 1 day of rehydration and found that its content was significantly reduced compared to LD treatment (**Figure S1E**), in which probably due to the activity of the P5CS is regulated by feedback inhibition or/and transcriptional factor under HD treatment (Yoshiba et al., 1995; Verslues and Bray, 2006; Silva-Ortega et al., 2008). Typically, Schafleitner et al. (2007) found at the early drought stress stage, the proline content and the activity of P5CS was coordinated but with the stress lasted, the P5CS was less abundant and the correlationship was also cut down, which meant there was other regulation mechanism. Notably, the proline accumulation was still higher in severe water-stressed treatment and made less connection with yield maintenance or biomass production. Moreover, Larher et al. (1993) observed that a strong accumulation of proline was caused by high external concentrations of sucrose and glucose. Whereas it was same that the sucrose and glucose pathway had changed, this was the other possible reason that the high accumulation of proline content observed under HD treatment (**Figure 2**). Very interestingly, we noticed that the FPKM value of *P5CS2* under HD treatment was significantly lower than HDCK since the normal proline content was housekeeping for plant normal development (Kaur and Asthir, 2015) (**Table S3**). Additionally, decreased expression of *P5CS2* under HD treatment had promoted more embryo lethal since the special role of *P5CS2* in embryo development. It was one possible explanation to the higher empty abortive rate under HD treatment (**Figure 1**; **Table 1**).

### 4.4 Regulator mechanism of gibberellic acid biosynthesis under drought stress

Gibberellic acid (GAs) belongs to a large group of tetracyclic diterpene carboxylic acids that act throughout the life cycle of plants by activating cell division and cell elongation mechanisms, stimulating their growth and development (Colebrook et al., 2014). Among the many gases synthesized by plants, GA1 and GA4 are the main bioactive forms (Sponsel and Hedden, 2010). Although this class of hormones is primarily associated with stem elongation, seed germination and reproductive development in plants, it has been shown to be involved in plant tolerance to drought stress. Early evidence for the involvement of GA in abiotic stress tolerance comes from the observation that the application of growth retardants confers drought resistance to plants by inhibiting the endogenous synthesis of GA (Rademacher, 2000).

Most evidence emphasizes that dioxygenases are involved in regulating GA biosynthesis and that GA2ox genes respond primarily to abiotic stresses in plants (Yamaguchi, 2008; Hedden and Thomas, 2012; Binenbaum et al., 2018). According to the results of RNA-seq, the genes expression encoding GA20ox had up-regulated, an enzyme that controls the synthesis of gibberellin, and GA2ox, an enzyme for gibberellin degradation, mostly down-regulated under LD treatment. However, under HD treatment, the expression of GA20ox slightly changed but the expression of GA2ox significantly increased (**Table S3**). It inferred that GA is dynamically regulated by the equilibrium of biosynthesis and deactivation rate, while genes encoding GA2-oxidase under stress condition is up-regulated by stress responding signal in many species (Hedden and Kamiya, 1997; Magome et al., 2008). Shi et al. (2019) found that transgenic plant with up-regulated expression of *GhGA2ox1* had exhibited higher drought tolerance with higher free proline and relative water content by up-regulating *GhP5CS*, *GhDREB1* and *GhWRKY5*. Moreover, overexpression of *AtGA2ox1* gene in maize had not only increased proline and soluble sugar content, but also had deactivated bioactive of GA and inhibited biosynthesis pathway of GA (Chen et al., 2019). In foxtail millet, it was same that the expression of genes encoding GA2ox was up-regulated and the fold change was higher than that of GA20x under LD and HD treatment, which meant the deactivation rate of GAs was speed up. It was notable that the expression of GA2ox had induced semi-dwarf phenotype of *Arabidopsis*, which was counteracted by application of bioactive GA3 with normal shoot length (Lee et al., 2014). Although the rehydration indeed happened after HD treatment, the plant height (**Figure S2**) and the panicle length of foxtail millet were abridged under HD treatment, which served as one reasonable causes of condensed plant height and panicle length (**Figure 1**). More importantly, over-expression of GA2-oxidases had frequently led to changes of floral morphologies and/or loss of fertility (Singh et al., 2002). This was verified in rice by using constitutive promoter with drawf phenotype and loss of fertility (Sakamoto et al., 2003). Similarly, the empty abortive rate, especially under HD treatment, was increased by compromising yield as to increased up-regulated expression of genes encoding GA2ox both in LD and HD (**Table 1**). The other important thing was that the genes relating to GA20ox was up-regulated under LD treatment, which gave one opportunity for stem and panicle elongation (**Figure 1**). It is possible to manipulate GA20-oxidase gene expression to modify plant height (Hedden and Phillips, 2000). Zhou and Underhill (2015) found that a cohort of GA20-oxidase genes, and two of them, *AaGA20ox1* and *AaGA20ox3*, was of perspective role as dwarfing targets of mutagenesis to regulate endogenous levels of GA to control stem elongation. Alleles of GA20-oxidase gene had promoted normal stem by negatively worked on mutant gene of short stature *sd1* in rice but also promoted normal flower formation since the active GAs were key regulator for floral process (Sasaki et al., 2002). Consequently, the sustainable prominent strategy of dealing with drought is to maintain bioactive GAs by exogenous GA_4_/GAs for the bi-benefits of panicle length and floral formation/fertility.

## 5 Conclusion

**Figure 10:**
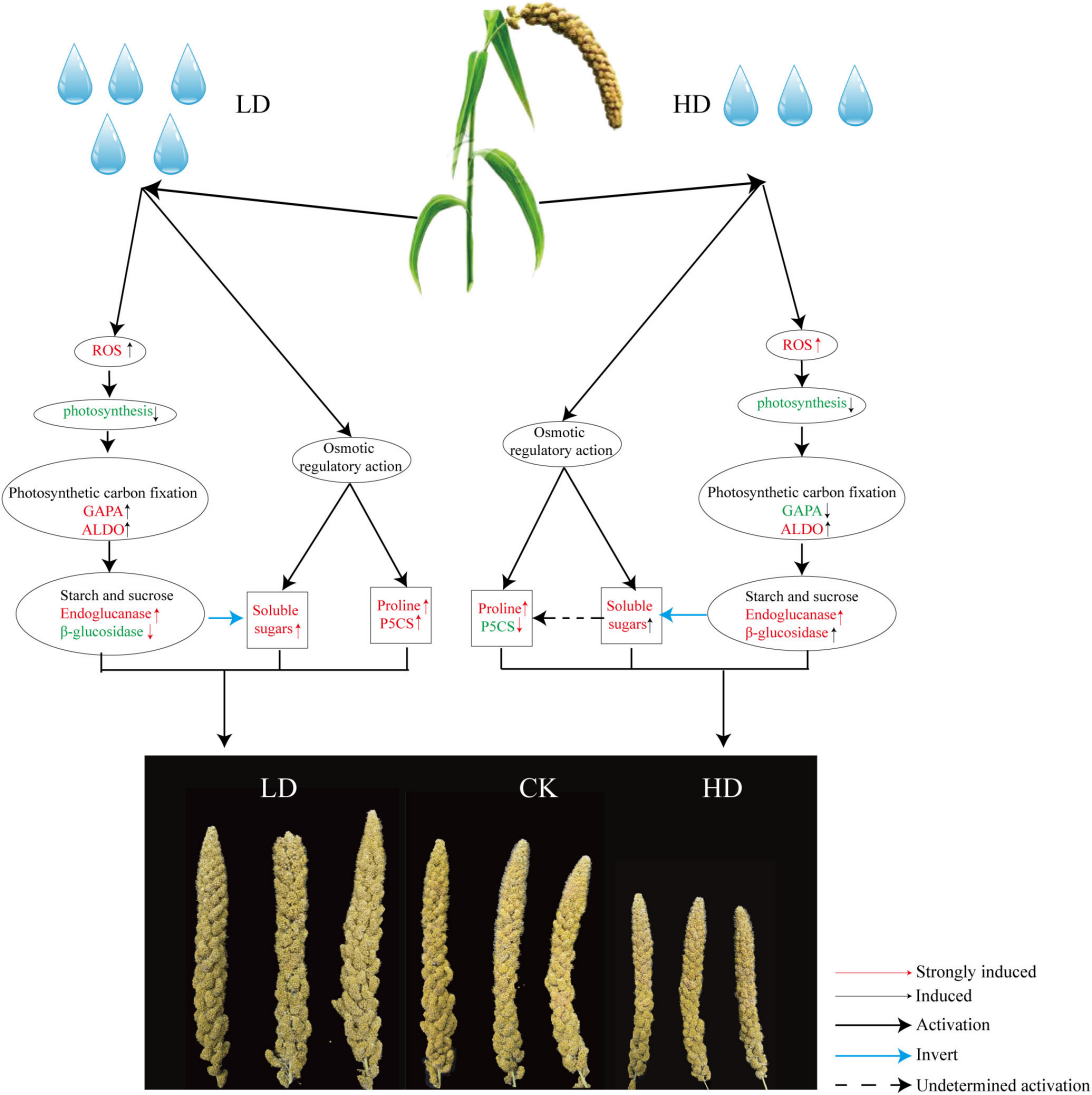
Regulation model of foxtail millet under drought stress at yield formation stage and the key pathway including phytohormones, antioxidants, phenylpropanoid synthesis pathways, osmotic regulatory pathways, and photosynthetic carbon fixation pathways. Red font indicates increased and up-regulated change. Green font indicates decreased and down-regulated change.

Consequently, one working model had been proposed to describe the effects of drought on the yield development stage of foxtail millet (**Figure 10**). When foxtail millet was exposed to drought stress, the stress induced the production of ROS, including O^2-^ and H_2_O_2_. Excessive accumulation of ROS had caused oxidative damage to plant cells, resulting in the reduction photosynthesis of foxtail millet. Importantly, the reduction of photosynthesis had affected photosynthetic carbon fixation and starch and sucrose metabolism (Endoglucanase *β*-glucosidase decomposition) improving accumulation of soluble sugars in foxtail millet leaves and affected the development of millet under LD and HD treatment. And the excess accumulation of ROS in turn has improved an increase in antioxidant enzymes SOD and POD in foxtail millet leaves.

On the other hand, drought stress leads to osmotic pressure imbalance in foxtail millet, and the content of osmotic regulators such as soluble sugar (starch and sucrose biosynthesis) and proline (ariginine and proline metabolism) in leaves has also increased. The key path way of LD treatment is the change of starch and sucrose metabolism, phenylpropanoid biosynthesis, phenylalanine, tyrosine and tryptophan biosynthesis (**Figure 4**). The key path way of HD treatment is the change of carbon fixation in photosynthetic organisms, diterpenoid biosynthesis, phenylpropanoid biosynthesis (**Figure 4**). But under HD treatment the gene *P5CS* is down-regulated obviously thereby promoting the empty abortive rate of foxtail millet. Moreover, GA decompositon (GA2ox) has aslo been significantly lifted, especially in HD, which also has nectively effect on yield of millet by making more floral process failed. Overall, it can be concluded that either LD or HD treatment could induce molecular reprogramming of foxtail millet to responding to drought stress but at the expenses of yield.

## Data availability statement

The original contributions presented in the study are publicly available. This data can be found here: NCBI, PRJNA906029.

## Author contributions

JW and ZS have contributed equally in this paper. XNW has assisted with process of physiolotical index. YT, XL, and CR have participated in photosynthsis process. JR has given suggestion for GO and KEGG pathway drawing. XW and CJ have helped with yield index. CZ, SZ, and HZ have given suggestions for data processing. XBL, SK, and XZ have taken picture of plant. All authors contributed to the article and approved the submitted version.
