# Supplementary material for: Transcriptome-based analysis of key pathways relating to yield formation stage of foxtail millet under different drought stress conditions
Source: Front Plant Sci. 2023 Feb 3;13:1110910. doi: 10.3389/fpls.2022.1110910 (PMC9937063; doi:10.3389/fpls.2022.1110910)
Supplement: Supplementary file 1 [file DataSheet_1.docx]

Supplementary Material

Transcriptome-based analysis of key pathways relating to yield formation stage of Foxtail millet under different drought stress conditions

Jing Wang ^†^, Zexin Sun ^†^, Xinning Wang, Ying Tang, Xinyi Li, Chuanyou Ren, Jingyao Ren, Xiaoguang Wang, Chunji Jiang, Chao Zhong, Shuli Zhao, He Zhang, Xibo Liu, Shuli Kang, Xinhua Zhao, Haiqiu Yu*

*** Correspondence:** Haiqiu Yu ^*^

yuhaiqiu@syau.edu.cn

# Supplementary Figures and Tables

## Supplementary Tables

| Table S1 Primer sequences of candidate reference genes | | | | |
| --- | --- | --- | --- | --- |
| id | Symbol | Description | (F) Sense primer ( 5’- 3’) | (R) Antisense primer (5’- 3’) |
| LOC101765011 | EF1a | internal reference gene | TGACTGTGCTGTCCTCATCA | GTTGCAGCAGCAAATCATCT |
| LOC101765110 | AGPL3 | glucose-1-phosphate adenylyltransferase large subunit 3, chloroplastic | CCACCACTTTAACCGACAT | AGCCTGTAACATCCTCCAAT |
| LOC101775420 | P5CS2 | delta-1-pyrroline-5-carboxylate synthase 2 | AGAGGGGAAGGAACTGGT | ATACAGATGTCTCGAAGGAAGT |
| LOC101771288 | GLU3 | endoglucanase 12 | AAATAGTTGCTTGCTTTGTCTG | CTTCGTTTCTCACTTTCTCG |
| LOC101784482 | PSB28 | photosystem II reaction center PSB28 protein, chloroplastic | GGCACGGACGAGCAAACG | GCCGACGAGTCGAACACC |
| LOC101763965 | ADC2 | arginine decarboxylase 2 isoform X1 | ATGATGAATGGACGGGCAAAT | AACACGAAGCAAGTAAAGCA |
| LOC101756021 | ACS1 | aminocyclopropane-1-carboxylate synthase | CCTTCCTGTCCTGACGCATTT | CGGGTTGGAGGGGTTGGT |
| LOC101781059 | BGLU7 | beta-glucosidase 7 isoform X1 | GTGTCTTCTGTCGGAACCTA | CAAACCTGCATCAAATGTAATA |
| LOC101782181 | CPA | N-carbamoylputrescine amidase | GGTGAGAAATAATCATACGCTC | GGACAGGGAAAGATAGGGA |
| LOC101786844 | TPP9 | probable trehalose-phosphate phosphatase 9 | CAACGCCACCTGAACCCA | CGACGCTCTTCTTCTCCTAAC |
| LOC101784073 | CIN1 | beta-fructofuranosidase, cell wall isozyme | AGTCTGCAAGTTGACGGAA | CGAGGACGAGTTGGGGAT |

| Table S2 Information of RNA-seq performed for millet leaf tissue in the presence or absence of simulated drought treatment | | | | |  |
| --- | --- | --- | --- | --- | --- |
| Sample | Raw Data (bp) | Clean Data (bp) | Unique_Mapped (%) | Total_Mapped (%) | |
| LD-1 | 6532312800 | 6471626166 | 39132937 (90.52%) | 40087197 (92.73%) | |
| LD-2 | 5940495300 | 5880384767 | 36355211 (92.53%) | 37286385 (94.90%) | |
| LD-3 | 8773887900 | 8698418373 | 52701092 (90.76%) | 54417276 (93.71%) | |
| LDCK-1 | 7990060800 | 7910233723 | 47240966 (89.52%) | 48386937 (91.70%) | |
| LDCK-2 | 7836592500 | 7766286731 | 47096999 (90.87%) | 48216402 (93.03%) | |
| LDCK-3 | 7565417700 | 7492896340 | 45106508 (90.12%) | 46174062 (92.25%) | |
| HD-1 | 6998472900 | 6932166139 | 33969277 (73.22%) | 34732663 (74.87%) | |
| HD-2 | 7581267600 | 7510538144 | 44207586 (88.03%) | 45269551 (90.15%) | |
| HD-3 | 7009167300 | 6942190111 | 41890736 (90.30%) | 42880171 (92.43%) | |
| HDCK-1 | 6340152600 | 6286304748 | 37495653 (89.48%) | 38459460 (91.78%) | |
| HDCK-2 | 7112151900 | 7053012801 | 42424571 (90.19%) | 43485914 (92.45%) | |
| HDCK-3 | 6391366500 | 6331535817 | 38230501 (90.35%) | 39172058 (92.58%) | |

## Supplementary Figures


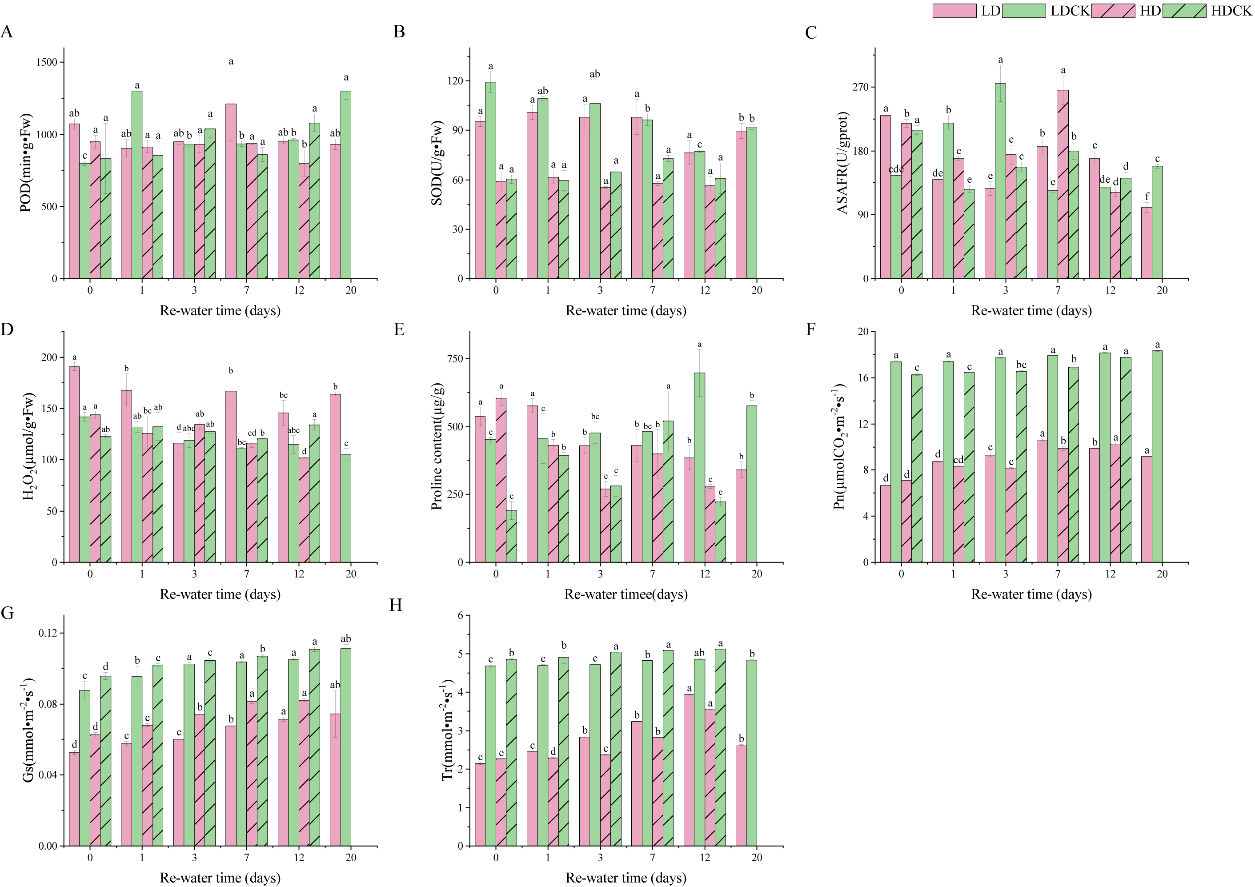


Fig S1. Antioxidant enzyme activity, ROS content, osmosis substances and photosynthetic characteristics of millet leaves after rehydration under different drought stresses. (A) POD content; (B) SOD content; (C) ASAFR unit; (D) H_2_O_2_ content; (E) Pro content; (F) Pn; (G) Gs; (H) Tr. Bars with different letters indicate significant differences between each treatment and the CK at the P=0.05 probability level. After 12 days under HD treatment, the millet is already for harvest. Therefore, the rehydration treatment after HD treatment is due to 12 days.


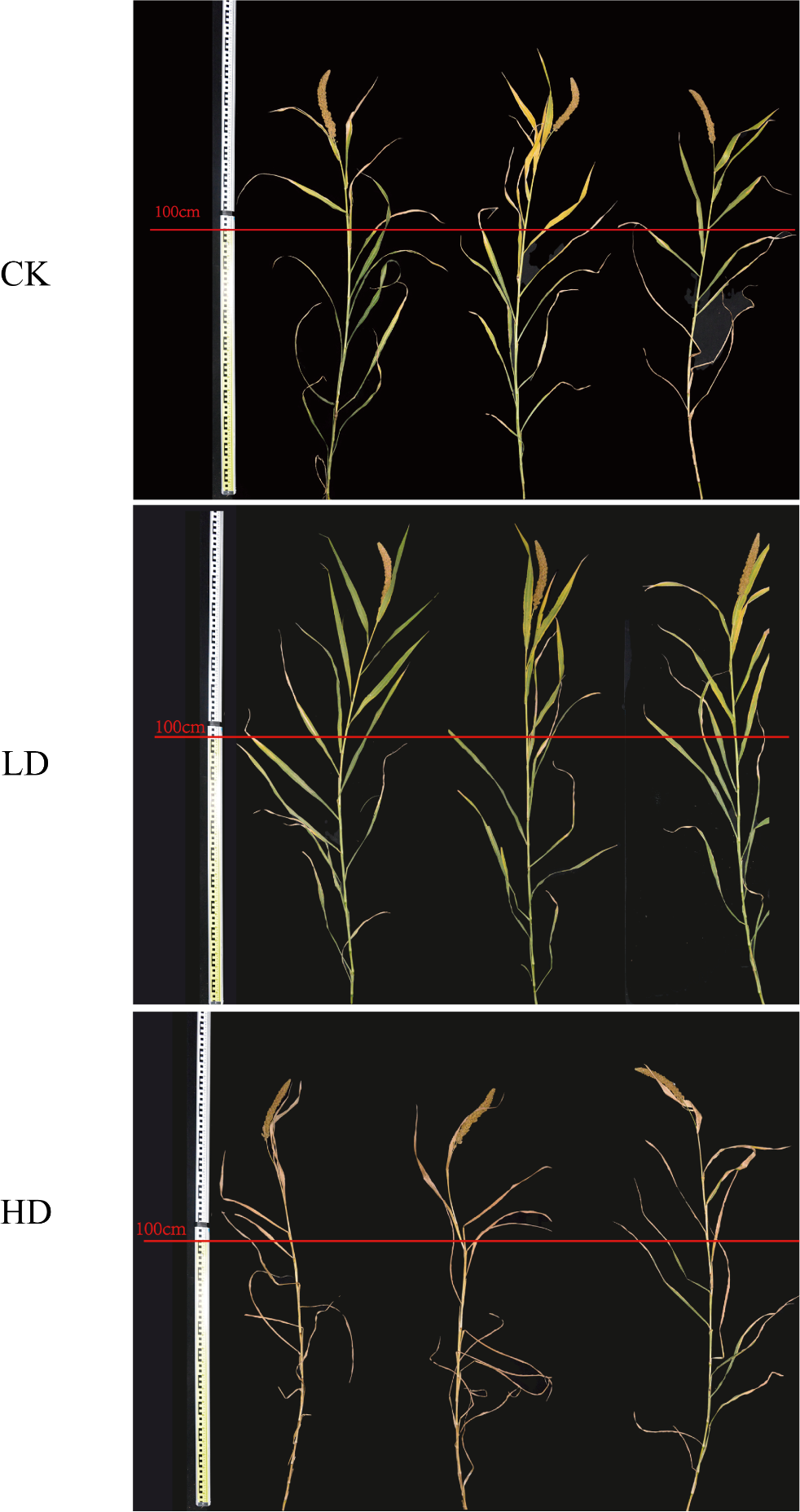


Fig S2.Morphological changes of plant height under LD, HD and CK. Due to the limitation of the size of the figure, a mark was made at 100cm of the ruler.
